# Supplementary material for: Trial-by-trial learning of successor representations in human behavior
Source: PLoS Comput Biol. 2025 Nov 17;21(11):e1013696. doi: 10.1371/journal.pcbi.1013696 (PMC12643320; doi:10.1371/journal.pcbi.1013696)
Supplement: Attachment — Submitted filename: Response to Reviewers.pdf [file pcbi.1013696.s002.pdf]

Response to reviewers for “Trial-by-trial learning of successor representations in human behavior”

Ari E. Kahn[1] Dani S. Bassett[2], Nathaniel D. Daw[1,3]

[1]Princeton Neuroscience Institute, Princeton University, Princeton, NJ, USA
[2]Department of Bioengineering, University of Pennsylvania, Philadelphia, PA, USA
[3]Department of Psychology, Princeton University, Princeton, NJ, USA

ABSTRACT

We would like to thank the editor and the reviewers for evaluating our manuscript “Trial-by-trial learning of successor representations in human behavior”, which we have revised in accordance with the reviewers’ suggestions and now resubmit for your consideration for publication in PLOS Computational Biology. In the pages that follow, we summarize our responses and the changes made point-for-point. For easier reference, reviewer comments have been consecutively numbered. New text in the manuscript has been highlighted in red, and author responses in blue. We thank the editor and the reviewers very much for the opportunity to incorporate these suggestions into our manuscript and resubmit our paper. Please do not hesitate to contact us if we can provide further clarification.

General note to all reviewers:

We have replaced the use of recency_ntrials (the number of trials since a node was last observed) with log(recency_ntrials) in all relevant analyses. While this parameter was not a focus of any of the analyses in the manuscript, we found that this led to significantly improved stability of model fitting, as well as improved fit assessed with IAIC. We have confirmed that all results of the original manuscript hold, though various estimates have slightly changed.

We have generated a temporary OSF link at https://osf.io/jupbz/overview?view_only=31849bb44e1e40d78f35f72577fcb3eb

Editor Comments :

While the reviews are positive in general, they also raise some major concerns, which need to be addressed. Some of the presentation of the Methods is overly concise, an appendix or supplement might help for issues the authors do not want to include in the main text, but which would be needed for replication. I in particular had trouble understanding the human experiments from the paper. I understand that the data is from a previous publication, but at least the group sizes (subjects per group) and the number of trials per subject would be useful and also help understanding whether the statistical tests conducted were appropriate. When I tried to access the OSF repository, I was required to send a personalized access request. While this would have been ok for me, it might compromise anonymity of the reviewers, so it cannot be expected that they consider this material. The authors should consider providing a hidden osf read-only link for review of a revision (and of course make the link public, once the manuscript is accepted) or double check that their repository is public.

Thank you very much for your consideration of the manuscript. While the number of trials was included in the methods, it is now mentioned in the results as well. The number of subjects included in the current analysis has also been added. Relevant sections are copied below:

Results:
These models were fit to reaction times across three sets of random walks, either on a modular (n=173), lattice (n=185) or random (n=177) graph, with each walk comprised of 1500 trials.

Methods:
We fit our models to participants from \textcite{Kahn2018_NetworkConstraintsLearnability}. All participants provided informed consent as specified by the Institutional Review Board of the University of Pennsylvania, and study methods and experimental protocols were approved by the Institutional Review Board.

Participants completed 1500 trials of a serial reaction time-like task, responding to a cue shown on the screen as rapidly as possible with a one- or two-button key combination. Unbeknownst to participants, the sequence of cues followed a traversal through one of various graph structures: either a modular graph composed of 15 nodes in 3 clusters, a lattice graph, or a randomly connected graph. Each subject was assigned two of the three graphs, and performed the task twice in sequence with counterbalanced ordering. Node sequences were generated via a random walk on the assigned graph. An additional subset of subjects performed the task on one of the three aforementioned graphs, followed by 500 trials on a fully connected graph. The assignment of key combination to node was randomized across subjects. We include the first set of trials for those subjects, but exclude the fully connected graph from our analysis. We excluded all trials on which subjects responded incorrectly, as well as trials with implausible RTs (those less than 30 ms). Across the two task stages of 1500 trials each, our analysis included 173 subjects for the modular graph, 185 subjects for the lattice graph, and 177 subjects for the set of random graphs.

We have additionally included a temporary OSF link at
https://osf.io/jupbz/overview?view_only=31849bb44e1e40d78f35f72577fcb3eb

Author Summary

Our ability to plan intelligently requires predicting the state of the world multiple steps into the future. Enumerating future outcomes step-by-step, however, is slow and costly. Instead, research has shown that people rely on simplified models of the world that skip across multiple steps at once. How do we construct these simplified models? One promising idea is the successor representation (SR), which predicts future events via a simple and neurally plausible computation. The SR has been shown to explain a range of behavioral phenomena, but these studies have not identified which among many learning rules the brain uses to build the SR. Plausible mechanisms for learning associations over delays (called bootstrapping and eligibility traces) both converge to identical simplified world models, and thus existing studies on the SR, which focus on well trained behavior, are unable to distinguish between them. Here, we answer this question by examining behavior on a graph learning task, where stimulus-by-stimulus reaction times have been shown to reflect predictions over long temporal horizons. Through both model fitting and model-agnostic comparisons, we find that behavior is best explained by a learning rule heavily dependent on eligibility traces, in contrast to previous work which generally assumed an (untested) bootstrapping update rule.

Reviewer #1:

In this manuscript, Kahn et al. present a series of model-based and model-agnostic analyses of previously collected data, in order to test how participants update expectations of upcoming stimulus-action requirements ("states"). They consider three main mechanisms through which participants may learn these expectations, being a "recency" mechanism, a "one-step" prediction mechanism, and a "successor representation" which is able to learn multi-step predictions of upcoming states.

The authors define a previously unconsidered model of recency and demonstrate that this is a major driver of human response times, making an important methodological contribution to the modeling of response times in graph learning tasks. They then demonstrate how multistep predictions can still further improve the model, suggesting successor representation-like learning does take place in these tasks.

Finally, the authors define two model-agnostic tests, to directly test the influence of eligibility traces and bootstrap updating, two learning mechanisms that may contribute to successor representation learning. They only find evidence for eligibility traces, but not for bootstrapping, questioning whether successor representation learning is based on vector prediction error signals as often considered in theoretical work. However, the authors note the limited amount of data available for the bootstrapping analysis compared to the trace analysis, and do not perform a test that can confirm the absence of an effect (e.g. a Bayes Factor analysis).

This work addresses an important research question, and I am particularly fond of the two model-agnostic signatures the authors were able to identify to test traces and bootstrapping. While I think the research question and results are of importance and fit the scope of Plos Computational Biology, there are several minor points that I think can be addressed to improve the strength of the current work, which I will list in order of importance:

1.

Perhaps any appendices to this paper were not communicated during this peer review process, but I was missing some proof of concept (a simulation study) that the parameters and models considered in this manuscript are indeed recoverable and distinguishable (cf. Wilson & Collins, 2019; https://doi.org/10.7554/eLife.49547).

We appreciate this suggestion from the reviewer. We have included two new analyses in the supplement. First, we have added simulation and recovery for parameters of interest, where we find that we can accurately recover the key parameters, namely lambda, gamma, and inverse softmax temperatures. We do so with the most complete model in the paper, including all recency effects. Second, we have included a confusability study where we simulate reaction times based on our recovered parameters, and then compare model fits between TD0, TD1, and TDλ. Both analyses are copied below.

Parameter Recovery

We wanted to verify that our model was capable of recovering distinct parameter regimes for parameters of interest. We verified parameter recovery through the following procedure. For each simulation, we chose a set of mean values for λ, γ, β_A, and β_W. We exhaustively simulated all combinations of λ in [0.1, 0.3, 0.5, 0.7, 0.9], γ in [0.5, 0.7, 0.9], β_A in [-2, -1, 0], β_W in [-2, -1, 0], treating each as the mean value of that parameter in our simulated dataset. For each of these 5 x 3 x 3 x 3 combinations of parameter values, we created a new set of 100 subjects. To create this set of subjects, we first sampled the best fit parameters for a single subject (from the experimental data), with replacement. Then, for that subject and for each of the four target parameters, we sampled the target parameter from a normal distribution centered at the target group mean with variance equal to the recovered group variance from our best fit model in the manuscript. We then generated synthetic reaction times, sampling log RTs from N(μ,σ), where μ is the predicted mean log RT from our simulation and σ is the value fit for that individual subject. This process was repeated for all 100 subjects. Finally, we re-fit the model and all free parameters to this new simulated dataset.

In figure S1, we plot the range of recovered group-level parameters across all simulations. We collapse across all parameter combinations: the recovered values of λ for λ=0.1 include all possible combinations of other parameters. We find that for all combinations of graphs and parameters, the recovered group-level parameters successfully captured the simulated means and were clearly distinguishable.

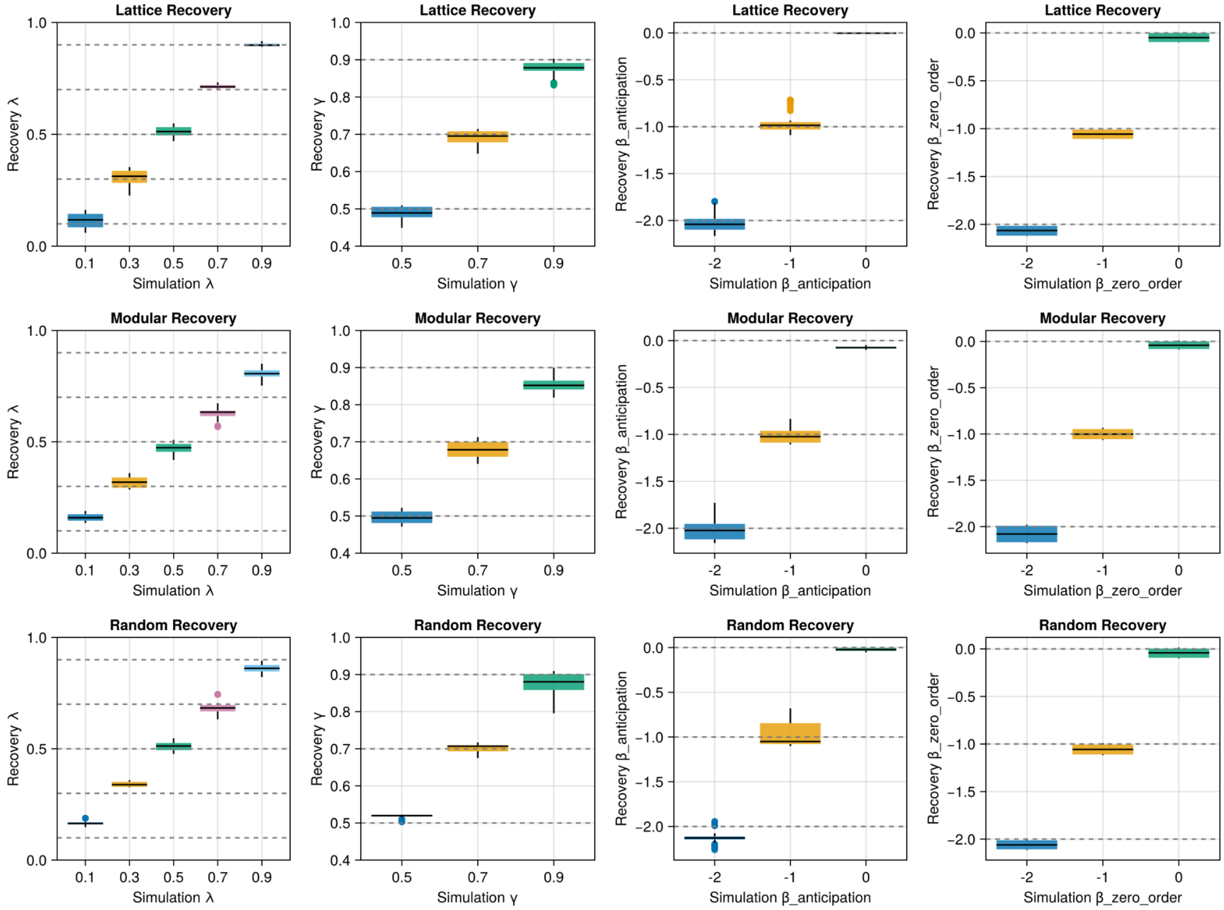

Figure S1: Parameter Recovery. Boxplots indicate median, 25th, and 75th percentiles, and whiskers indicate 1.5 * interquartile range. Outlying points are plotted separately.

Model Distinguishability

To test that our models were distinguishable and able to capture unique predicted sources of variance, we simulated RTs using a TD0, TD1, or TDλ model. For each simulated subject, we sampled best fit parameters of a single subject with replacement from a model fit to our experimental data, with either TD0, TD1, or TDλ, and used those parameters and the associated model to generate new RTs, sampling log RTs as in our parameter recovery. We then fit data from 100 such subjects, using either the TD0, TD1, or TDλ model. We generated 20 unique datasets for each combination of simulation and recovery model. Plotted in figure S2 are the differences in fit versus the best fitting model for each simulation, measured via lAIC. We found that, for each combination of simulation and recovery, recovery with the appropriate model led to the lowest lAIC score for 19/20 or 20/20 simulations in all conditions.

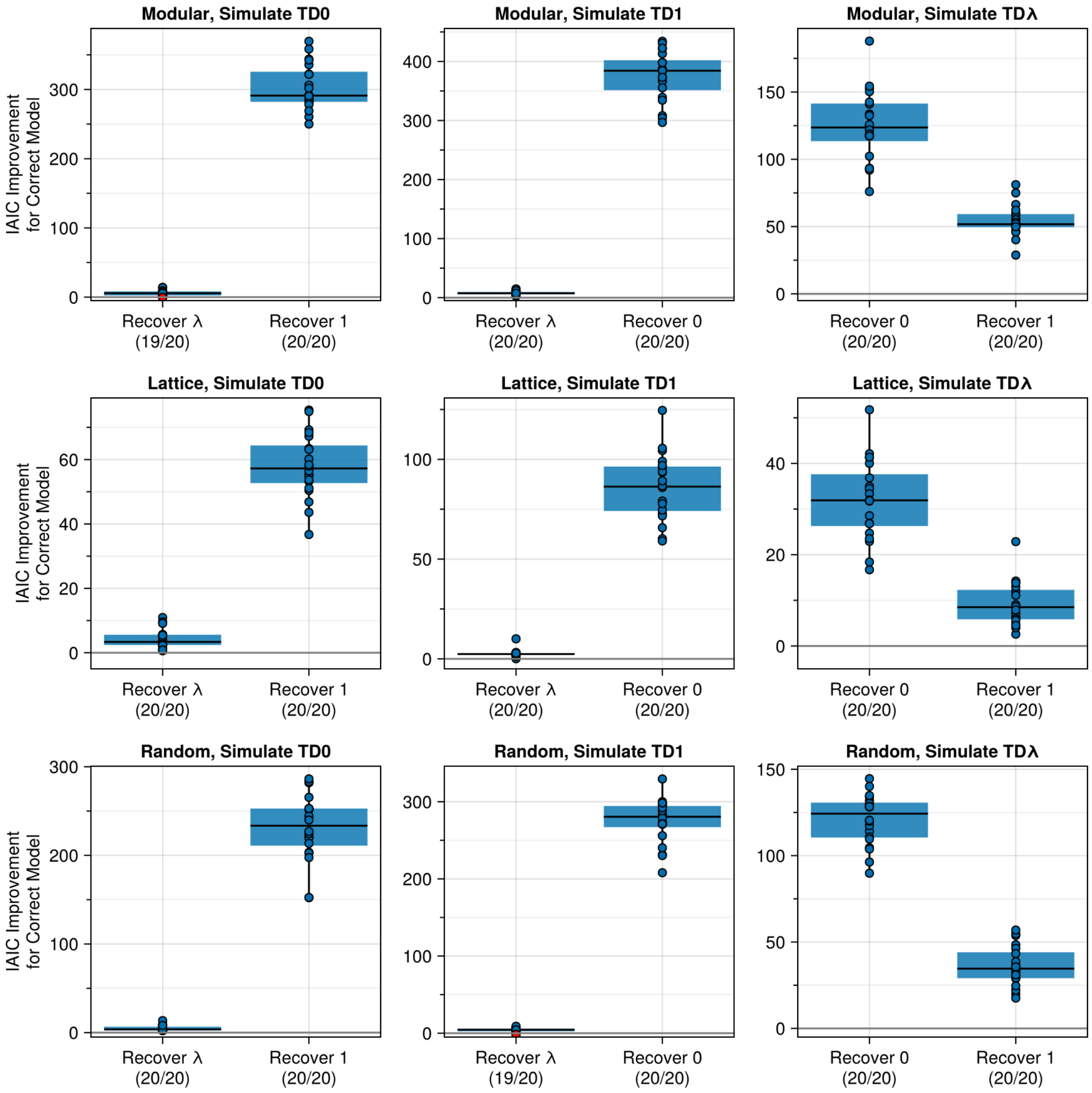

Figure S2: Model distinguishability. Each point is the improvement in lAIC for recovery with the data simulation model versus an alternative model. Boxplots indicate median, 25th, and 75th percentiles, and whiskers indicate 1.5 * interquartile range.

2.

Throughout the manuscript, there are several references to the idea that an SR-TD(lambda = 1) model corresponds to a Hebbian learning rule rather than a vector-prediction-error learning rule. However, this is not mathematically exposed in the main text, and is also not readily apparent to me based on the brief model description provided in the "SR-TD learning model" section of the methods. I think readers may find it very informative if this relationship is made more explicit, and it may inform some of the points that follow.

We appreciate the reviewer pointing out that this relationship may benefit from more explanation.

Consider an update rule with the following form:

```
M[s_t, :] *= (1 - alpha)
e[:] = gamma * e[:] + onehot(s_t)
M[:, :] += alpha * e[:] * onehot(s_t)
```

This is Hebbian (outer product) learning between a vector of (gamma-discounted) eligibility traces and a vector for the current state indicator; the correlation between such traces and state encounters is, by definition, the SR.

But it can also be motivated in a couple of other ways.

In particular, this rule can be derived from the TD(1) rule for M by noting that in the specific case when lambda=1, the last two terms in the TD error vector (onehot(s_t) + gamma M[s_t+1, :] - M[s_t, :]) – the bootstrap and error comparison terms – actually have essentially no effect, since the increment due to each bootstrap (+ gamma M) is exactly subtracted away by the error comparison (- M) term at the following timestep (since the state that was s_t+1 is now s_t and almost every state's eligibility decays by exactly gamma in the intervening step, when lambda=1). Thus, we can derive the Hebbian variant of the rule, as given above, by simply omitting these terms. (The decay at M[s(t),:] then arises from accounting for the state where e is incremented and so the last term;s decrement is not canceled.)

Another way to motivate this rule is to see that each time a state is encountered and its eligibility incremented, this triggers a series of updates for each subsequently encountered state s_t (each gamma-discounted via the decaying eligibility). These together, over steps, sum to a single Monte Carlo sample of the subsequent occupancy vector, and M[s_t,:] is thus ultimately updated by that sample with weight alpha; the aggregated effect is like an episode-level Monte Carlo update (1-alpha) * M[s_t,:] + alpha * sample_vector.

We thought it might be too much of an aside to go through all this in the current manuscript (though we are happy to take advice from the reviewer about this). We have added the following to the manuscript:

In particular, when $λ=1$, each bootstrap term is canceled on the next trial's update due to the trace itself decaying by exactly γ. This means it can be omitted and an equivalent update for each state can be computed using just the eligibility traces and the encountered states. This amounts to a series of scalar, element-wise updates for each state encounter weighted by each state's decaying eligibility, or equivalently Hebbian learning between the decaying eligibility traces and the encountered states.

3.

Further detailing this, I was wondering if the SR-TD(lambda = 1) model might mimic the "temporal context model" of the episodic memory system (Howard & Kahana, 2002; https://doi.org/10.1006/jmps.2001.1388), in which a trace of previous stimuli is maintained and associated with incoming stimuli. If this is true, the authors might cite this work to better contextualize this special case.

The reviewer insightfully asks about the relationship with the TCM (temporal context model), a longstanding memory encoding and retrieval model which successfully explains a number of observed phenomena in free recall. Indeed, it has previously been observed that TCM can be viewed as a case of the Successor Representation with a TD(lambda) learning rule (Gershman et al. 2012; Zhou et al 2025). Strictly speaking, this relationship holds exactly only for the classic TCM scenario of a single presentation of each stimulus. The TCM as originally written diverges from TD on the second encounter of a state/stimulus (though this is not a typical situation in memory studies): in part, due to the emergence of a nonzero contribution from the bootstrap on the second encounter.

Although Gershman et al. (2012) viewed the bootstrap effect as a substantive differentiating prediction of the SR model with TD(lambda) learning, , we agree with the reviewer that TD(1) comes closer to the original vision of TCM since it can be learned with a fully Hebbian rule, albeit with an additional decay term that was not included in the original TCM. (This is a point we also made, if somewhat obliquely, in Zhou et al., 2025). However, while we think all these considerations clarify a space of possible mechanisms and models (and the present experiment investigates their contributions, albeit not in a list learning scenario), since they all mostly coincide in the classic case of a single presentation per item, we are not sure that the previous work really intended a strong commitment to, e.g., the strictly Hebbian case.

We have included discussion of this relationship:

Such a Hebbian learning rule would also relate to another prominent model of human memory, the TCM (Temporal Context Model) \autocite{Howard2002_DistributedRepresentationTemporal}. The TCM was developed to explain a number of phenomena consistently observed in free recall, including recency and contiguity effects. Interestingly, the TCM can be understood as learning the SR \cite{Gershman2012_SuccessorRepresentationTemporal,zhouEpisodicRetrievalModelbased2023} so long as stimuli are fully unique, but the two diverge upon any repetition (such as re-visiting the same node in the current study). Specifically, under certain constraints, the context vector in the TCM functions identically to the eligibility trace in the SR, where the first visit to state j will set the prediction from i to j to e_i. Due in part to nonzero contribution of bootstrapping, the TD(lambda) rule for learning an SR differs from classic TCM on the second encounter \cite{Gershman2012_SuccessorRepresentationTemporal}, whereas a purely Hebbian (eligibility trace only) variant of TD(1) SR learning stays closer to the original conception of TCM. Unlike classic list learning experiments (which typically present each item only once), the present experiments visit each graph node many times, exercising the differences between these various learning approaches, which largely coincide on the first visit. Of course, it remains to be tested whether similar conclusions (e.g. that learning is best explained by a combination of traces and bootstrap, but primarily the former) would be seen in analogous experiments testing memory recall, in experiments with multiple item encounters.

4.

If SR-TD(lambda = 1) is indeed such a special case, I think it is worth it to consider a variant of SR-TD in which lambda is not a free parameter, but instead fixed to 1, and to see if there is a significant improvement when lambda is allowed to be free. This would at least expose the data as containing variance that goes beyond the simple associative mechanism of the temporal context model. Currently, the authors interpret the fact that their parameter estimates do not yield this extreme value of lambda = 1 as circumstantial evidence of this, but I don't believe this is an effective way to interpret the model fit. Instead, I think it is worth statistically formalizing this point with an explicit model comparison. A lack of significance then would further question the involvement of the vector prediction error for learning multistep predictions.

This is an excellent point. We have included a formal model comparison test between SR-TD(lambda) and SR-TD(1). We find that SR-TD(lambda) provides a significantly better fit when compared to SR-TD(1) for all three graphs, suggesting that while eligibility traces play a much larger role than previously assumed, it does appear that some additional variance is accounted for by bootstrapping-like mechanisms.

To test whether eligibility traces alone explained learning, we first verified that a model with freely varying λ should be distinguishable from one with λ fixed at extreme values of 0 or 1 (Fig. S2). We then compared subject-level marginal likelihoods (see Model Comparison) from our model fits with λ as a free parameter to those where λ was fixed at 1, and found that allowing it to freely vary significantly improved model fits in all cases (Paired t-tests, one-sided $t_{172}=3.32$, 95\% CI: 0.14 to 0.55, $p < 0.002$ (modular), $t_{184}=5.57$, 95\% CI: 0.15 to 0.31, $p<0.001$ (lattice), $t_{176}=3.68$, 95\% CI: 0.14 to 0.46, $p<0.001$ (random)). This suggests, in contrast to previous work, that learning is predominantly driven by eligibility traces, though we do not rule out bootstrapping altogether.

5.

In the case of SR-TD(lambda = 1), does the discount factor gamma become redundant (because learning is Hebbian and not error-driven)?

The discount factor does not become redundant, because gamma determines the asymptotic target of learning rather than just something transient about the learning update. Gamma determines the ‘target’ of how far into the future the SR is predicting, or how quickly the trace is decaying in the case of TD1. Hebbian learning correlates the state encounters with the gamma-discounted traces, and (like all the learning rules) it has the fixed point inv(I - gamma *T), which of course depends on gamma. Alternatively, consider that while TD1 /can/ be implemented without error-driven learning, it’s entirely equivalent to doing so with error-driven learning and a decay of the trace only dependent on gamma, but here gamma will still show up in the error-driven target.

6.

Could the authors point out a qualitative difference between a pure recency learning model, and a model that includes multi-step predictions? This seems to be a major point of the manuscript, and such a difference might be identifiable in the modular graph for example by considering transitions within communities as in Figure 5 of Wientjes & Holroyd (2024; https://doi.org/10.1371/journal.pcbi.1011312).

The reviewer is asking for a qualitative prediction unique to the SR when compared to a pure recency model, which we agree would significantly strengthen the manuscript. One such prediction relates to entry into a new cluster. The SR predicts that the reaction time on the first node in a new cluster should be slower than the second node – because conditional upon cluster entry, the SR expects the new node over and above any effects of recency. Visualizing this effect requires carefully controlling for any effects of recency. Previous approaches have observed similar phenomena by contrasting individual RT pairs, such as in Karuza et al. 2017. Here we instead analyzed first and second RTs following entry to a new cluster as a function of recency. To eliminate additional sources of variance, we first fit a reduced model that hierarchically accounted for baseline RT, RT shift, motor movements and effects of trial (i.e. time on task), but excluded all recency and learning mechanisms. We then fit a hierarchical linear model of the form residual log RT ~ recency + (1st or 2nd node in cluster). Note that we exclude a recency of lag 1 for the first node, as this is not possible for the second node in the cluster (and corresponds to an edge case of leaving and immediately returning to the same cluster). We find a significant effect of node type, as reported below:

\subsection*{SR Learning Exists Alongside Recency Learning}

To further verify that our effects were not solely explained by recency, we examined a prediction of the SR in the modular graph which is not shared by a recency learning model, the difference between first and second trials upon entry to a new cluster. For the modular graph, the SR assigns a lower predictive weight to cross-cluster transitions than to transitions within the same cluster. However, once a new cluster is entered, the SR now will exhibit a bias towards predicting nodes in the new cluster. On average, this suggests that the first node should exhibit a slower RT than the second node. We fit a mixed effects linear model to residual log RTs after accounting for motor response and trial number, and included recency as a categorical control (see Methods). We found that the second trial was indeed significantly faster than the first (β = -0.029, SE = 0.013, t(102.0) = -2.33, p = 0.02), confirming that learners exhibit behavior predicted by the SR.

\subsection*{Novel Cluster Analysis}

To test whether participants exhibited slower responses on the first node upon entering a new cluster than the second, we fit a linear mixed effects model to residualized RTs. These residualized RTs were derived by fitting a model of the form of our learning models, but which only included effects of trial number and motor response. We then found all trials that were either the first or second entry into a new cluster. We fit a model of the form $resid\_rt \sim time\_since\_node + cluster\_step + (1 + cluster\_step|subject))$, where $cluster\_step$ was either 0 for the first node or one for the second, and $time\_since\_node$ was the number of trials since the node was last seen, for all lags up to 100. $time\_since\_node$ fit as a categorical with an effects coding scheme such that $cluster\_step$ was relative to the grand mean. Models were fit $MixedModels$ v4.24.1 \autocite{MixedModels} in $Julia$ 1.10.4 \autocite{Julia-2017}.

7.

In the "combined models" section, the formula for mean response times no longer seems to include the

ntrials and lag10 regressors. I missed a motivation for dropping these regressors from the model and wonder if some of the associated variance might not be wrongly assigned to the successor representation regressor now, potentially influencing the estimates of lambda and gamma.

Thank you for catching this omission in the methods. We did in fact include these two regressors in the combined model, but the terms were mistakenly dropped in the manuscript. The manuscript has been updated to include these.

We further found that the lag10 regressor was causing instability in the model fits, due to its potentially unbounded growth. We found that replacing lag10 with log(lag10) uniformly improved both the fitting process as well as fits themselves (as assessed via IAIC), and have updated the methods to note that:

Additionally, a simpler recency model was added in-line with previous work by including regressors $\beta_{ntrials}$ and β_{lag10}, which respectively were applied to the number of trials elapsed since node s_t last occurred, and the log of the number of occurrences of node s_t within the last 10 trials.

8.

As a purely optional and supplementary suggestion, the authors might consider whether their estimation of successor representation-related parameters (lambda, gamma) show any reliability for these participants, for example by correlating estimates of an odd-even split of the trials (contribution to the likelihood) of a single graph learning task, or even considering the parameter values of two different graph learning tasks (for the participants that completed two different graphs). Especially, if estimates of the discount factor gamma show some reliability, this could maybe be taken as evidence that prediction error-driven learning (bootstrapping?) does contribute some variance to these data.

We appreciate this suggestion to investigate within-subject reliability. While the current study was not explicitly designed around such questions and our arguments and results do not turn on individual differences, the ability to parameterize individual subject behavior is indeed a promising direction for future work. We should say at the outset that we are somewhat skeptical of testing reliability using split-halves over timeseries data (eg due to potential subtle violations of IID) and would be more confident doing so with explicit test-retest data. A second reason we are cautious is that we are estimating model parameters in a hierarchical model using expectation maximization and a Laplace approximation, and while we have used this extensively for baseline model estimation, we can't vouch for its accuracy estimating reliability (where the top level of the hierarchy is more complicated since it aims to estimate the covariance between the splits). In any case, the results we present below appear promising, but we decided not to include them in the article due to these uncertainties.

We implemented the odd-even split of trial likelihood contribution suggested by the reviewer. To quantify reliability, we computed the test-restest reliability approach from Rouder & Haaf 2019, which explicitly estimates reliability via estimating the covariance between per-split random effects in a hierarchical model. In brief, for each free parameter in our original model, x, we fit two free parameters in our augmented model, x_1 $(mean)$ and x_2 $(difference)$. For even and odd splits, learning proceeded over all trials, with likelihood only incorporated either odd or even trials, and likelihood was summed across the two splits. The value of x in split 1 was $x_1 + x_2$, and the value of x in split 2 was $x_1 - x_2$. Test-retest reliability was then given by $\rho = \sigma_1^2 - \sigma_2^2/\sigma_1^2 + \sigma_2^2$, where σ_1 and σ_2 were the group-level variances of x_1 and x_2.

Below are the results of this preliminary reliability analysis. We combined data across the three graphs, and computed the reliability separately for the first and second stages of data. Group-level variances are plotted on the left for both the mean and difference terms. Parameter reliability is plotted on the right. Reliability for gamma is $\rho = 0.901$ for stage 1, and $\rho = 0.9$ for stage 2. We hope to further investigate the reliability of individual differences in future work.

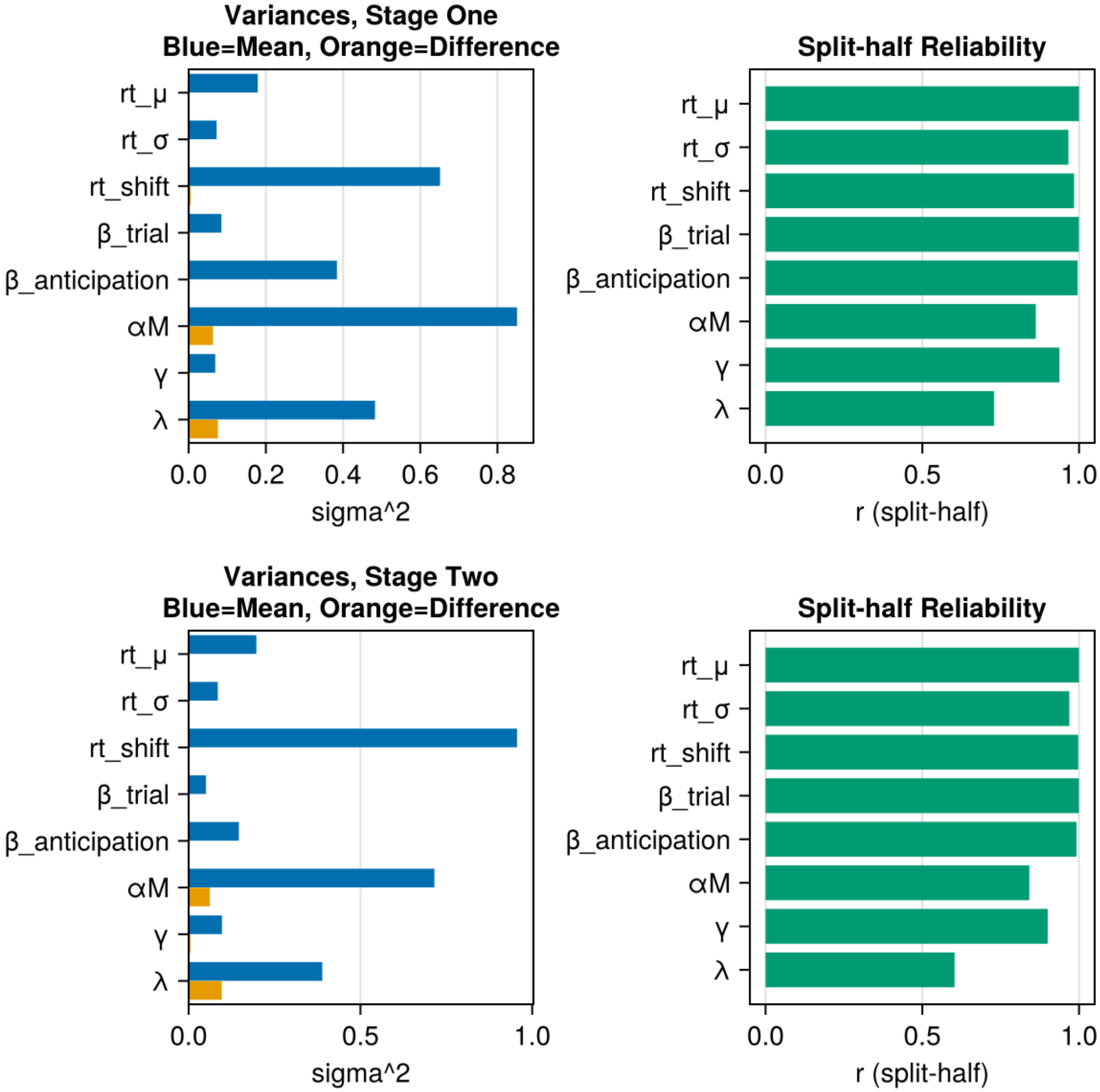

9.

Some minor typographical errors:
In the methods section "recency learning model", the beta(lag10) is not stylized into a greek letter.
In the method section describing the "bootstrap analysis", it is described to identify an enhanced RT on the final step of "SR". I believe this should refer to "ST", being the last transition of the bootstrap sequence, rather than the abbreviation for the Successor Representation (SR; causing me some confusion initially).

We greatly appreciate the reviewer catching these typographical errors.

Reviewer 2

Reviewer #2: In this study, the authors examine response times in a serial reaction time task in which the stimuli trace out edges of a graph with one of three structures. Of particular interest, the modular graph has a temporal community structure as introduced in work by Anna Schapiro and colleagues. In previous work (e.g., Lynn, Papadopoulos, Kahn, & Bassett, 2020, Nat Physics) it was shown that human RTs are slower across community boundaries compared to within community. This finding is counterintuitive from a certain perspective; although in general RT is predicted by the entropy of the transition, the entropy of transitions within boundary are the same as across boundaries. Note that across-community edges are differentiated by powers of the transition matrix, this paper pursues the hypothesis that the successor representation (SR), which asymptotes as exponentially-weighted powers of the transition matrix, predicts human RTs. The idea is intuitively appealing; rather than ``moving'' along the true transition matrix, human learners ``move'' along the SR. The authors compare the SR to other simple models. By comparing specific sequences they can also evaluate whether the SR is updated by temporal difference learning or by simple aggregation of eligibility trace memories.

1.

There are some substantial methodological points (below) that speak to some of the empirical basis for the conclusions about the models, but these are relatively minor. The major result, which is very likely to be sound, is that a recency-biased model carries more variance in RT than the SR model. The observation of a recency effect here does not really get much attention (recency isn't part of standard SR) but it's pretty interesting. There are certainly extremely robust recency effects in (all kinds of) memory experiments with random lists of stimuli. Recency effects among responses are also quite widespread in RT tasks (sequential dependencies in simple choice RT tasks have been well-characterized since at least the mid-1970s). However, recency bias is not universally observed in SRT tasks**. For instance Gureckis & Love (2010, Cog Sci) observed an ``anti-recency effect'' in an SRT task when stimuli were less likely than chance to repeat at short lags. Perhaps the recency effect in this dataset reflects some kind of learning about the statistical structure rather than simply decaying activations.** Additional control analyses could possibly tease this out from the existing data. Perhaps a recency bias reflects an prior about the statistics of the world (Anderson & Schooler, 1991, Psych Sci; Gershman, et al., 2014, PLoS Comp Bio)?

We thank the reviewer for their supportive feedback. The reviewer points out that the recency effect here (both its form and overall contribution) is very much of interest, and perhaps we have not given it sufficient attention in the manuscript. While the current manuscript focuses on the SR more than the recency effect mostly due to the authors having conceived the current study as a means to investigate SR learning, a possible relationship between the recency effect and higher-order statistical models is indeed quite interesting and an idea that we hope to investigate further in future studies.

Regarding the recency rule the reviewer brings up an interesting point: that the learning rule for recency here, which is an exponentially decaying activation, matches up quite closely with the task itself. It may be that people have a prior or innate expectation of a recency effect, but it’s also quite possible that people quickly recognize in the context of the current study that prior occurrence of a response is an effective predictor of future occurrences of that same response, and tune their responses as such. Both of these

seem quite plausible, and the idea that people intrinsically tune the forms of these statistical models is intriguing. We have added mention in the discussion.

While statistical patterns akin to this recency effect are ubiquitous in the natural world (generally speaking, the recent past is an excellent predictor of the future), people are capable of learning other patterns such as an ``anti-recency effect'' \autocite{gureckisDirectAssociationsInternal2010} after sufficient exposure. Why, then, do we specifically see such strong recency behavior on the current task? One possibility is that people rely on priors which suggest the form of recency we observe, and unlike \textcite{gureckisDirectAssociationsInternal2010}, are not given evidence to the contrary. Another is that they are quickly adapting to the observed task statistics. Such adaptation would likely be at an extremely short timescale. Indeed, we do observe a much larger improvement in reaction times at the start of the task, but this is likely confounded by learning the task response paradigm itself. In the future, we hope to test to what degree this recency model is drawn from prior experience versus learned directly from the statistics of the task at hand.

That being said, we think the correct way to do this would be to design a study in which (like Gureckis & Love) we directly manipulated such zeroth-order recency statistics. It doesn't strike us as feasible to test in the current dataset, given that any experience driving recency models is going to be quite similar across participants, and presumably the basic form of repetition in this study becomes clear very early in. In particular, we currently ignore the likelihood contribution of the first 500 trials for each subject, and any learning of the form of recency in this task is likely masked by early effects of familiarity with the response paradigm. Perhaps more subtly, given that the SR here is fitting to higher-order statistics, particular care would need to be taken to control how predictable these manipulations are when conditioning expectations on the current state as in the SR.

2.

The other interesting result is that, by comparing RTs on specific motifs, the results favor a model in which the SR is constructed by updating with the current memory trace rather than bootstrapping via temporal difference learning. So, what to make of all this in the context of existing modeling on SR? Well, if the eligibility trace is allowed to cue subsequent states in addition to the SR, this would account for the recency effect. In models of laboratory memory, the exponentially-decaying eligibility trace is sometimes referred to as a temporal context vector (Gershman, ... Sederberg, 2012, Neural Comp). And the SR constructed from trace update rather than bootstrapping (and with $\gamma=0$) is (more or less) the temporal context model. **Is this account falsified by the data? Presumably one would need to disentangle the exponential decay over powers of the transition matrix from γ and the exponential decay due to λ. In the temporal community graph at least, these seem to be perfectly confounded with one another, no?**

The reviewer raises a question of the relationship between the SR (and particularly the SR+recency) versus the TCM. Indeed, multiple papers including Gershman et al. have shown that the SR and TCM are quite deeply related, leaving aside for a moment the addition of the recency effect here. This is with the caveat, at least we understand it, that the equivalence between the models only strictly holds for the first presentation of a stimulus. The flip side of this is that – on our understanding, at least, the classic TCM story for list learning isn't highly theoretically committed or data-constrained as to what happens on the second encounter, so all the variants we consider (which all basically coincide for the first encounter) might be seen as reasonable extrapolations of TCM-like learning to the current task.

In response to the reviewer's specific questions: First, the model with gamma=0 would lead to T(I-0*T) = T, the single-step predictive model, which is already included and tested against in the manuscript.

Second, lambda and gamma are, we believe, distinguishable in our current study. On a model level, while the two both relate to exponentially decaying processes, gamma determines asymptotic properties of the learned representation, while lambda solely affects the transient learning process, leading to distinct behavioral predictions. The extreme case of gamma=0 would lead to unidentifiability of lambda as well, via the combined effect of lambda*gamma on the trace. Fortunately, outside this case, the two are separately recoverable, as we find in the recoverability analysis now added to the supplement and copied below.

Parameter Recovery

We wanted to verify that our model was capable of recovering distinct parameter regimes for parameters of interest. We verified parameter recovery through the following procedure. For each simulation, we chose a set of mean values for λ, γ, β_A, and β_W. We exhaustively simulated all combinations of λ in [0.1, 0.3, 0.5, 0.7, 0.9], γ in [0.5, 0.7, 0.9], β_A in [-2, -1, 0], β_W in [-2, -1, 0], treating each as the mean value of that parameter in our simulated dataset. For each of these 5 x 3 x 3 x 3 combinations of parameter values, we created a new set of 100 subjects. To create this set of subjects, we first sampled the best fit parameters for a single subject (from the experimental data), with replacement. Then, for that subject and for each of the four target parameters, we sampled the target parameter from a normal distribution centered at the target group mean with variance equal to the recovered group variance from our best fit model in the manuscript. We then generated synthetic reaction times, sampling log RTs from $N(\mu,\sigma)$, where μ is the predicted mean log RT from our simulation and σ is the value fit for that individual subject. This process was repeated for all 100 subjects. Finally, we re-fit the model and all free parameters to this new simulated dataset.

In figure S1, we plot the range of recovered group-level parameters across all simulations. We collapse across all parameter combinations: the recovered values of λ for λ=0.1 include all possible combinations of other parameters. We find that for all combinations of graphs and parameters, the recovered group-level parameters successfully captured the simulated means and were clearly distinguishable.

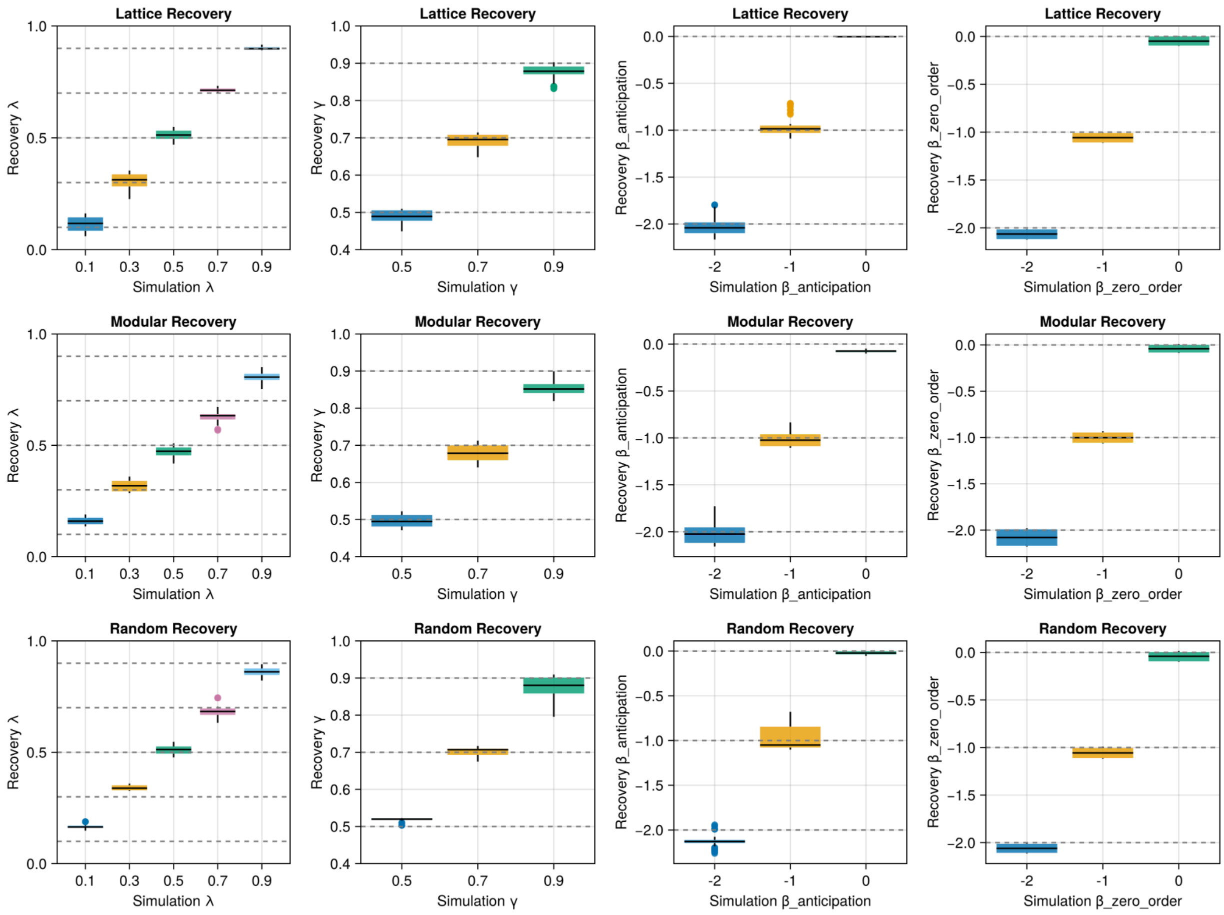

Figure S1: Parameter Recovery. Boxplots indicate median, 25th, and 75th percentiles, and whiskers indicate 1.5 * interquartile range. Outlying points are plotted separately.

Finally, perhaps the reviewer's main insight is that the functional form of the trace and of the recency effect themselves are quite similar. We find this quite an intriguing idea but also somewhat counterintuitive, in the sense that from the RL perspective, the trace plays quite a different role, i.e. in gating learning rather than itself serving as a cue, whereas the idea that it might itself also serve as a predictive cue (and thus account for recency as well) seems a bit odd, though perhaps it is more natural from the TCM perspective. In any case, we agree that (whatever its motivation), this is a testable model.

The update rule via a learning rate for the recency effect is mechanistically identical to the effect of gamma and lambda on the trace - both set the current state to one after decaying the entire vector by a constant. While quite preliminary, we fit a model which predicted recency via the trace. This principally requires yoking both to have the same decay parameter, but note that this is not *just* that - the update rule for recency was fixing the entire vector to sum to one (via its update of decaying the entire vector by (1-alpha) at each step, while the eligibility trace does not have a similar constraint. We have included below the results of this model alongside the others currently in the manuscript. However, at least in this form, we find it to significantly underperform compared to a model that allows separate trace and recency mechanisms (modular: $t(172)=-3.73$, $p<0.0002$, lattice: $t(184)=-5.39$, $p<2.133$, random: $t(176)=-6.17$, $p<4.55$). The idea of finding a unified form of these systems may nonetheless be an interesting direction to investigate in the future.

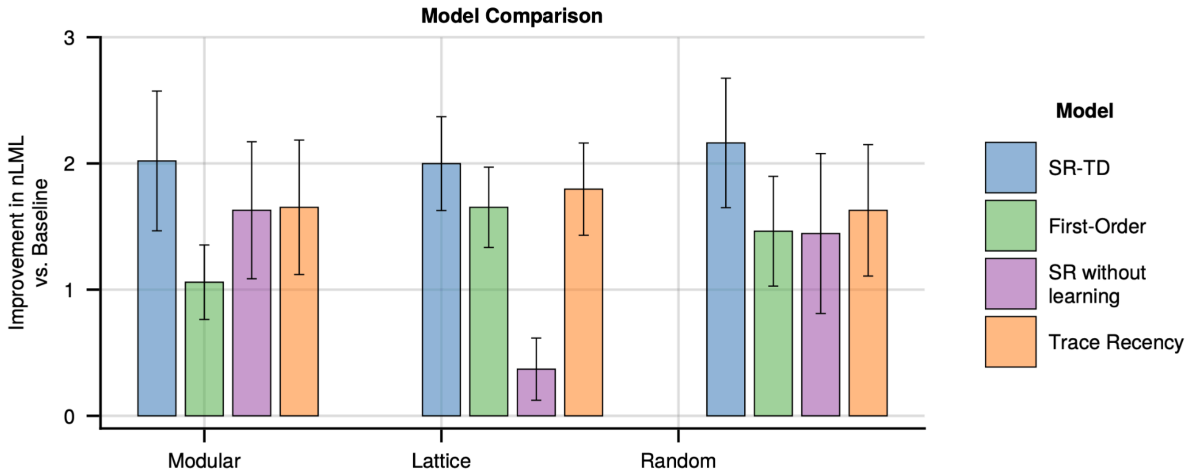

3.

The paper would be strengthened a great deal by making the connection between the recency+trace SR model and temporal context models. This class of models has been extensively studied in laboratory memory tasks (although not any SRT papers that come to mind).

We have added discussion of the relationship between the models, as we believe the close relationship will certainly be of interest to readers:

Such a Hebbian learning rule would also relate to another prominent model of human memory, the TCM (Temporal Context Model) \autocite{Howard2002_DistributedRepresentationTemporal}. The TCM was developed to explain a number of phenomena consistently observed in free recall, including recency and contiguity effects. Interestingly, the TCM can be understood as learning the SR \cite{Gershman2012_SuccessorRepresentationTemporal,zhouEpisodicRetrievalModelbased2023} so long as stimuli are fully unique, but the two diverge upon any repetition (such as re-visiting the same node in the current study). Specifically, under certain constraints, the context vector in the TCM functions identically to the eligibility trace in the SR, where the first visit to state j will set the prediction from i to j to e_i. Due in part to nonzero contribution of bootstrapping, the TD(lambda) rule for learning an SR differs from classic TCM on the second encounter \cite{Gershman2012_SuccessorRepresentationTemporal}, whereas a purely Hebbian (eligibility trace only) variant of TD(1) SR learning stays closer to the original conception of TCM. Unlike classic list learning experiments (which typically present each item only once), the present experiments visit each graph node many times, exercising the differences between these various learning approaches, which largely coincide on the first visit. Of course, it remains to be tested whether similar conclusions (e.g. that learning is best explained by a combination of traces and bootstrap, but primarily the former) would be seen in analogous experiments testing memory recall, in experiments with multiple item encounters.

4.

Expository point:

Methods/results should be fleshed out and, at least high level methods should be embedded. (If there's a supplement/appendix with this information, it didn't make it to this reviewer.) As it is, there are a bunch of results missing that make it difficult to evaluate the paper's empirical contribution and what can really be learned from the modeling.

We apologize for any confusion in the methods. While the general approach (data source, modeling approach) were present, we have included additional detail that we hope is clarifying.

We have updated the description of the data as follows:

Participants.

We fit our models to participants from \textcite{Kahn2018_NetworkConstraintsLearnability}. All participants provided informed consent as specified by the Institutional Review Board of the University of Pennsylvania, and study methods and experimental protocols were approved by the Institutional Review Board.

Participants completed 1500 trials of a serial reaction time-like task, responding to a cue shown on the screen as rapidly as possible with a one- or two-button key combination. Unbeknownst to participants, the sequence of cues followed a traversal through one of various graph structures: either a modular graph composed of 15 nodes in 3 clusters, a lattice graph, or a randomly connected graph. Each subject was assigned two of the three graphs, and performed the task twice in sequence with counterbalanced ordering. Node sequences were generated via a random walk on the assigned graph. An additional subset of subjects performed the task on one of the three aforementioned graphs, followed by 500 trials on a fully connected graph. The assignment of key combination to node was randomized across subjects. We include the first set of trials for those subjects, but exclude the fully connected graph from our analysis. We excluded all trials on which subjects responded incorrectly, as well as trials with implausible RTs (those less than 30 ms). Across the two task stages of 1500 trials each, our analysis included 173 subjects for the modular graph, 185 subjects for the lattice graph, and 177 subjects for the set of random graphs.

We have added the following description of parameter estimation

\subsection*{Parameter Estimation}

We optimized the free parameters of the learning algorithms by embedding each of them within a hierarchical model to allow parameters to vary between subjects or simulated sessions. Subject-level parameters were modeled as arising from a population-level Gaussian distribution over subjects. We estimated the model, to obtain best fitting subject- and group-level parameters to minimize the negative log likelihood of the data using an expectation-maximization algorithm with a Laplace approximation to the session-level marginal likelihoods in the M-step \autocite{huysDisentanglingRolesApproach2011}

I don't see values of \beta_trial, \beta_{ntrials}, \beta_10 reported in the paper. One can learn a lot about whether the estimates of these parameters change across models (they should not). More broadly, the data should be reported in more detail. How do distributions of parameters across participants look? Do parameters trade off with one another?

We had originally excluded estimates of certain parameters that were not core to the analysis for the sake of clarity. However, we agree that these might be quite useful to understand, and we have included all subject-level parameter estimates to the supplement.

We have additionally included plots of subject-level parameter correlations in the supplement, which we hope will be informative.

Minor Methodological points:

5.

There are some claims/suggestions that could be explicitly tested to be on solid empirical ground. For instance, ``Interestingly on the average over participants, we estimate a remark- ably consistent degree of temporal discounting across all three graph topologies.." To make this observation one should test the model that supports this inference. The γ horizon seems to be about as big as could be sustained with these graphs, no? To seriously test the hypothesis, perhaps one can fix γ to be the same for all three graphs and compare that to the (nested) model with the γ's allowed to vary freely?

We agree that this claim was not adequately tested. A nested model comparison as suggested by the reviewer would be the optimal approach here, though we have had some computational difficulties optimizing all three graphs simultaneously (as necessary to test the nested hypothesis with a shared lambda). For the moment, we have rephrased our statement in the manuscript to simply mention the range of values observed, and note that the question of whether gamma varies across graph topologies would be a fruitful direction of future work. We hope that the revision copied below is satisfactory.

Alongside the parameter λ, which governs how long eligibility persists for learning over temporal delays, we also estimate a second parameter, γ, which controls how far into the future state occupancy predictions accumulate. In choice tasks, γ has long been of interest because it controls intertemporal preferences, i.e., how reward amounts trade off against delays. In the current task (which involves no choices), the parameter instead governs the timescale of implicit predictions as reflected in RT facilitation. Interestingly on the average over participants, we estimate all values falling between $γ=.717$ and $γ=0.894$, equivalent to a temporal horizon of 3.5 to 9.4 timesteps. An interesting question for future work is whether the predictive model we measure in RTs is also used for choice, e.g. if subjects learn a graph and are subsequently asked to plan routes or choose between states for reward \autocite{bornsteinCorticalHippocampalCorrelates2013,wientjesSuccessorRepresentationSubserves2024}. In this latent learning case, γ estimated from RTs might reflect individual differences that also play out in intertemporal choice. Alternatively, γ might be tuned to particular environments, e.g. the degree to which certain topologies enable longer-range prediction.

6.

All of the analyses in the paper model the effect of various variables on mean log RT. This is not unreasonable, but relies on an implicit assumption about the form of the RT distributions. Mean log RT is a pretty good proxy for drift rate in an evidence accumulation framework. But in at least some circumstances expectation is carried by non-decision time (Tiganj et al., 2022, JEP:G). And in at least some circumstances, recency is carried by the non-decision time (Bright, et al., 2022, bioRxiv). **A change in non-decision time across conditions would not only change mean log RT but also \sigma**. Tracking this down may turn out to be intricate but one could ask simple questions in an exploratory way. For instance, examination of RT distributions as a function of recency, or within/across-cluster transitions (etc) could be informative. **Another straightforward approach would be to explicitly allow sigma as**

well as mu to vary for different variables. Those models are nested. As an aside, the shape of the RT distributions associated with the entropy effect (from the 2020 Nat Rev Phys paper) are also very interesting.

We agree that this is quite interesting, though not the primary focus of the current manuscript. However, we do want to ensure that the current model is at least sufficient to capture the structure of learning via reaction times. To address this, we have examined whether our RT distributions vary as a function of recency. We relied on the recency values from our model (that is, the trace-like recency learning rule), and separated all trials into five equally-sized groups, based on those values. Shown are the residuals from our SR + Recency model fitting. Notably, there appear to be no obvious differences between the quintiles.

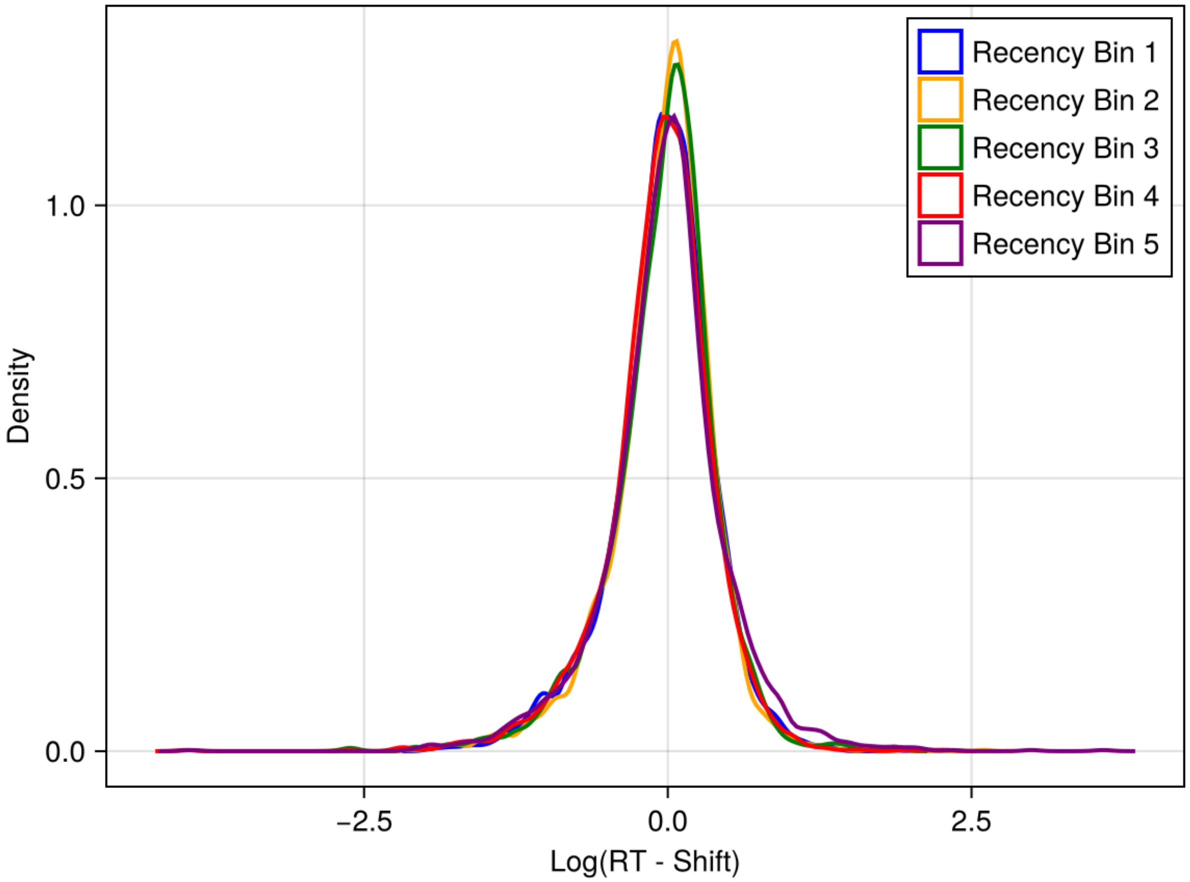

We have also added the following to our limitations:

In the current study, we are inferring anticipatory behavior from RTs, and doing so via a model in which variables linearly contribute to a log RTs. However, expectation can be influenced by non-decision time \cite{tiganjScanningCompressedOrdered2022}, as can recency \cite{brightTimeInitiateRetrieval2022}, and such changes to non-decision time are not fully captured by our model. Future work could better characterize such trial-to-trial dynamics through a process model of RTs that includes both decision and

non-decision times.

7.

Expository point related to the neural understanding of SR:
The discussion writes: ``Alternatively, neurons representing eligible states might be reactivated after a delay, via history stored in some separate memory system '' True, but there is also very strong evidence for slowly changing neural activation expressed in firing rates. There is pretty good evidence for exponential decay of firing, as predicted by eligibility trace representations (e.g., Danskin et al., 2023, Sci Adv), in a variety of regions in a variety of tasks. However, unlike the standard RL account there appears to be a continuous spectrum of time constants across neurons within the population. Wouldn't Hebbian learning with a multiscale eligibility trace give a multi-scale SR?

We thank the reviewer for pointing us in this direction. Indeed, there are quite a few plausible mechanisms for maintaining an eligibility trace - the number of such mechanisms (in contrast to a vector bootstrap signal) further fuel our interest in the learning rule for the SR. Differing decay constants also, as the reviewer mentions, provide a natural mechanism to learn relationships over different temporal scales. While the current task likely pushes subjects to model the world at a specific scale (that of single nodes or transitions), such multi-scale learning almost certainly has an important role in more naturalistic situations.

We have added the following points to our discussion:

Traces could also be subserved by persistent patterns of activity, as exponentially decaying neuronal firing is observed in the cortical neurons \autocite{danskinExponentialHistoryIntegration2023}. Such firing is thought to express a continuous range of time constants, which could provide a basis for learning SRs across multiple temporal scales \autocite{momennejadPredictingFutureMultiscale2018}.
